# 5-Carb­oxy-1,3-bis­(carb­oxy­meth­yl)-4-imidazolinium-4-carboxyl­ate

**DOI:** 10.1107/S160053681105416X

**Published:** 2011-12-23

**Authors:** You-Ming Zhang, Jian-Jun Ming, Jian-Peng Dang, Wan-Qiang Zhang, Tai-Bao Wei

**Affiliations:** a College of Chemistry and Chemical Engineering, Key Laboratory of Eco-Environment-Related Polymer Materials of the Ministry of Education, Gansu Key Laboratory of Polymer Materials, Northwest Normal University, Lanzhou 730070, People’s Republic of China

## Abstract

The title compound, C_9_H_8_N_2_O_8_, was obtained by the reaction of imidazole-4,5-dicarb­oxy­lic acid and 2-chloro­acetic acid. An intra­molecular O—H⋯O hydrogen bond occurs. The crystal packing is stabilized by intermolecular O—H⋯O and C—H⋯O hydrogen bonds, which link mol­ecules into a three-dimensional network.

## Related literature

The title compound is a potential polydentate ligand for the construction of metal-organic frameworks. For applications of metal-organic frameworks, see: Gao *et al.* (2005 ▶); Gurunatha *et al.* (2008 ▶); Wang *et al.* (2010 ▶); Zhang & Li (2010 ▶). For related structures, see: Chai *et al.* (2010 ▶); Liu *et al.* (2004 ▶); Lu *et al.* (2006 ▶).
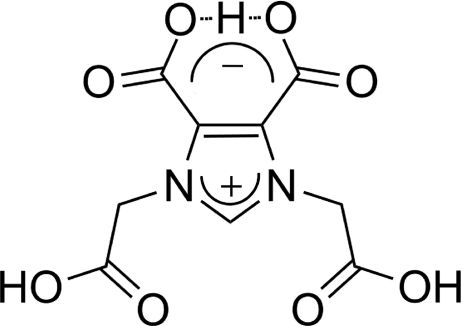


## Experimental

### 

#### Crystal data

C_9_H_8_N_2_O_8_
*M* *_r_* = 272.17
Orthorhombic, 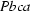

*a* = 8.986 (7) Å
*b* = 7.041 (6) Å
*c* = 33.68 (3) Å
*V* = 2131 (3) Å^3^
*Z* = 8
Mo *K*α radiation
μ = 0.15 mm^−1^
*T* = 296 K
0.35 × 0.33 × 0.29 mm

#### Data collection

Bruker APEXII CCD diffractometer
Absorption correction: multi-scan (*SADABS*; Bruker, 2008 ▶) *T*_min_ = 0.948, *T*_max_ = 0.957
13878 measured reflections
2091 independent reflections
1750 reflections with *I*2s(*I*)
*R*_int_ = 0.055

#### Refinement

*R*[*F* ^2^ > 2σ(*F* ^2^)] = 0.048
*wR*(*F* ^2^) = 0.109
*S* = 0.98
2091 reflections
185 parameters
H atoms treated by a mixture of independent and constrained refinement
Δρ_max_ = 0.23 e Å^−3^
Δρ_min_ = −0.19 e Å^−3^

### 

Data collection: *APEX2* (Bruker, 2008 ▶); cell refinement: *SAINT* (Bruker, 2008 ▶); data reduction: *SAINT*; program(s) used to solve structure: *SHELXS97* (Sheldrick, 2008 ▶); program(s) used to refine structure: *SHELXL97* (Sheldrick, 2008 ▶); molecular graphics: *SHELXTL* (Sheldrick, 2008 ▶); software used to prepare material for publication: *SHELXTL*.

## Supplementary Material

Crystal structure: contains datablock(s) I, global. DOI: 10.1107/S160053681105416X/rz2687sup1.cif

Structure factors: contains datablock(s) I. DOI: 10.1107/S160053681105416X/rz2687Isup2.hkl

Supplementary material file. DOI: 10.1107/S160053681105416X/rz2687Isup3.cml

Additional supplementary materials:  crystallographic information; 3D view; checkCIF report

## Figures and Tables

**Table 1:** Hydrogen-bond geometry (Å, °)

*D*—H⋯*A*	*D*—H	H⋯*A*	*D*⋯*A*	*D*—H⋯*A*
O3—H3*W*⋯O2	1.13 (3)	1.29 (3)	2.426 (3)	177 (3)
C1—H1⋯O6^i^	0.93	2.47	3.158 (3)	131
C4—H4*A*⋯O5^ii^	0.97	2.38	3.311 (3)	160
C4—H4*B*⋯O6^i^	0.97	2.37	3.046 (3)	126
C6—H6*A*⋯O7^i^	0.97	2.42	3.136 (4)	130
C6—H6*B*⋯O8^iii^	0.97	2.44	3.346 (3)	154
O5—H2*W*⋯O1^iv^	0.92 (3)	1.67 (3)	2.581 (3)	170 (3)
O8—H1*W*⋯O4^v^	0.93 (4)	1.84 (4)	2.710 (3)	155 (3)

Symmetry codes: (i) 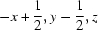; (ii) 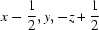; (iii) 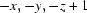; (iv) 
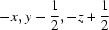
; (v) 
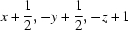
.

**Fig. 1.**
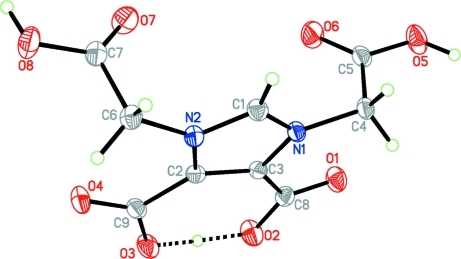
The structure of the title compound, showing the atom labelling scheme. Displacement ellipsoids are drawn at the 30% probability level.

## supplementary crystallographic information

### Comment

In recent years, increasing attention has been paid to the imidazole carboxylate ligands such as imidazole-4,5-dicarboxylic acid (H_3_IDC), (Gao *et al.*, 2005; Gurunatha *et al.*, 2008) and 1,3-dicarboxymethyl acid imidazolium (HDAM), (Wang *et al.*, 2010; Zhang *et al.*, 2010). H_3_IDC has six potential donor atoms (4O, 2 N) and can be partially or fully deprotonated to generate H_2_IDC^-^, HIDC^2-^ and IDC^3-^ anions at different pH values. Therefore, it can coordinate with metal ions in multi-coordinated ways to form a large diversity of supramolecular architectures (Lu *et al.*, 2006; Liu *et al.*, 2004). The zwitterionic dicarboxylate ligand HDAM, due to the presence of –CH_2_– spacers between the imidazole and carboxylate groups, has many degrees of flexibility and conformational freedom by bending or rotation when coordinating to the metal centre to give entanglements, conformational polymorphism and supramolecular isomerism, which may provide more possibility for the construction of unprecedented connected topological frameworks (Chai *et al.*, 2010). Taking the above into consideration, we designed and synthesized the title compound (H_3_DDAM) as a novel ligand, and its molecular structure is reported herein.

The molecular structure of the title compound is shown in Fig. 1. The values of  the C–O bond length within the carboxylic groups in 4- and 5-position of the imidazole ring range from 1.216 (3) to 1.295 (3), suggesting a delocalization of the negative charge within the two groups. As a consequence, the H3W proton is nearly symmetrically shared by the O2 and O3 oxygen atoms. The O1/O2/C8 and O3/O4/C9 carboxylic groups are approximately co-planar with the imidazole ring (dihedral angles 9.02 (14) and 10.37 (13)°, respectively), whereas the O5/O6/C5 and O7/O8/C7 groups are almost perpendicular, forming dihedral angles of 80.2 (2) and 88.1 (2)°, respectively. In the crystal structure, intermolecular O—H···O and C—H···O hydrogen bonds (Table 1) link molecules into a three-dimensional network.

### Experimental

Imidazole-4, 5-dicarboxylic acid (1.56 g, 0.01 mol) was slowly added to the stirred aqueous solution of 2-chloroacetic acid (2.82 g, 0.03 mol) and sodium hydroxide (1.2 g, 0.12 mol) in 30 ml of distilled water. The mixture was stirred for *ca* 4 h at reflux temperature, and the pH of the solution was controlled in the range of 8–11 with 5 *M* NaOH solution. Aqueous HCl (12*M*) was poured into the resultant mixture until the pH was 2–3. After cooling to room temperature, red block crystals suitable for X-ray structure analysis were obtained (2.28 g, yield: 83.7%). M.p.: 234–236 °C. Anal. Calcd for H_3_DDAM: C, 39.68; H, 2.94; N, 10.29, Found: C, 39.61; H, 2.98; N, 10.22.

### Refinement

The carboxylic H atoms were located in a difference Fourier map and refined freely. All other H atoms were included in calculated positions and refined in a riding-model approximation with C—H distances ranging from 0.93 to 0.97Å and *U*_iso_(H) = 1.2 *U*_eq_(C).

### Crystal data

**Table:**

C_9_H_8_N_2_O_8_	*D*_x_ = 1.697 Mg m^−^^3^
*M**_r_* = 272.17	Melting point: 510 K
Orthorhombic, *P**b**c**a*	Mo *K*α radiation, λ = 0.71073 Å
Hall symbol: -P 2ac 2ab	Cell parameters from 5190 reflections
*a* = 8.986 (7) Å	θ = 2.4–27.8°
*b* = 7.041 (6) Å	µ = 0.15 mm^−^^1^
*c* = 33.68 (3) Å	*T* = 296 K
*V* = 2131 (3) Å^3^	Block, red
*Z* = 8	0.35 × 0.33 × 0.29 mm
*F*(000) = 1120	

### Data collection

**Table:**

Bruker APEXII CCD diffractometer	2091 independent reflections
Radiation source: fine-focus sealed tube	1750 reflections with *I*2s(*I*)
graphite	*R*_int_ = 0.055
φ and ω scans	θ_max_ = 26.0°, θ_min_ = 2.4°
Absorption correction: multi-scan (*SADABS*; Bruker, 2008)	*h* = −11→11
*T*_min_ = 0.948, *T*_max_ = 0.957	*k* = −8→8
13878 measured reflections	*l* = −40→41

### Refinement

**Table:**

Refinement on *F*^2^	Secondary atom site location: difference Fourier map
Least-squares matrix: full	Hydrogen site location: inferred from neighbouring sites
*R*[*F*^2^ > 2σ(*F*^2^)] = 0.048	H atoms treated by a mixture of independent and constrained refinement
*wR*(*F*^2^) = 0.109	*w* = 1/[σ^2^(*F*_o_^2^) + (0.0398*P*)^2^ + 2.3475*P*] where *P* = (*F*_o_^2^ + 2*F*_c_^2^)/3
*S* = 0.98	(Δ/σ)_max_ < 0.001
2091 reflections	Δρ_max_ = 0.23 e Å^−^^3^
185 parameters	Δρ_min_ = −0.19 e Å^−^^3^
0 restraints	Extinction correction: *SHELXL97* (Sheldrick, 2008), Fc^*^=kFc[1+0.001xFc^2^λ^3^/sin(2θ)]^-1/4^
Primary atom site location: structure-invariant direct methods	Extinction coefficient: 0.065 (3)

### Special details

**Table:**

Geometry. All e.s.d.'s (except the e.s.d. in the dihedral angle between two l.s. planes) are estimated using the full covariance matrix. The cell e.s.d.'s are taken into account individually in the estimation of e.s.d.'s in distances, angles and torsion angles; correlations between e.s.d.'s in cell parameters are only used when they are defined by crystal symmetry. An approximate (isotropic) treatment of cell e.s.d.'s is used for estimating e.s.d.'s involving l.s. planes.
Refinement. Refinement of *F*^2^ against ALL reflections. The weighted *R*-factor *wR* and goodness of fit *S* are based on *F*^2^, conventional *R*-factors *R* are based on *F*, with *F* set to zero for negative *F*^2^. The threshold expression of *F*^2^ > σ(*F*^2^) is used only for calculating *R*-factors(gt) *etc*. and is not relevant to the choice of reflections for refinement. *R*-factors based on *F*^2^ are statistically about twice as large as those based on *F*, and *R*- factors based on ALL data will be even larger.

### Fractional atomic coordinates and isotropic or equivalent isotropic displacement parameters (Å^2^)

**Table:**

	*x*	*y*	*z*	*U*_iso_*/*U*_eq_	
C1	0.0333 (2)	0.0455 (3)	0.36566 (6)	0.0294 (5)	
H1	0.0683	−0.0768	0.3610	0.035*	
C2	−0.0421 (2)	0.3049 (3)	0.39610 (6)	0.0259 (5)	
C3	−0.0421 (2)	0.3386 (3)	0.35616 (6)	0.0254 (5)	
C4	0.0345 (2)	0.1384 (3)	0.29562 (6)	0.0298 (5)	
H4A	−0.0453	0.1904	0.2796	0.036*	
H4B	0.0406	0.0030	0.2906	0.036*	
C5	0.1801 (3)	0.2329 (3)	0.28505 (6)	0.0347 (5)	
C6	0.0369 (2)	0.0165 (3)	0.43809 (6)	0.0309 (5)	
H6A	0.0273	−0.1192	0.4338	0.037*	
H6B	−0.0339	0.0537	0.4584	0.037*	
C7	0.1931 (3)	0.0624 (3)	0.45163 (6)	0.0322 (5)	
C8	−0.0809 (2)	0.5139 (3)	0.33270 (6)	0.0312 (5)	
C9	−0.0854 (2)	0.4261 (3)	0.43069 (6)	0.0311 (5)	
N1	0.00421 (19)	0.1725 (2)	0.33789 (5)	0.0266 (4)	
N2	0.00520 (19)	0.1186 (2)	0.40108 (5)	0.0267 (4)	
O1	−0.0529 (2)	0.5150 (2)	0.29686 (4)	0.0433 (5)	
O2	−0.1404 (2)	0.6492 (2)	0.35153 (5)	0.0467 (5)	
O3	−0.1520 (2)	0.5842 (2)	0.42221 (5)	0.0436 (5)	
O4	−0.0606 (2)	0.3734 (2)	0.46442 (4)	0.0417 (4)	
O5	0.2115 (2)	0.2377 (3)	0.24692 (5)	0.0528 (6)	
O6	0.2616 (2)	0.2993 (3)	0.30957 (5)	0.0522 (5)	
O7	0.28441 (18)	0.1355 (3)	0.43067 (5)	0.0459 (5)	
O8	0.2134 (2)	0.0069 (3)	0.48852 (5)	0.0479 (5)	
H2W	0.149 (4)	0.170 (5)	0.2306 (9)	0.075 (10)*	
H1W	0.306 (4)	0.045 (5)	0.4978 (10)	0.082 (11)*	
H3W	−0.149 (4)	0.617 (4)	0.3891 (9)	0.073 (9)*	

### Atomic displacement parameters (Å^2^)

**Table:**

	*U*^11^	*U*^22^	*U*^33^	*U*^12^	*U*^13^	*U*^23^
C1	0.0302 (11)	0.0273 (11)	0.0306 (11)	0.0000 (9)	0.0007 (9)	−0.0047 (9)
C2	0.0233 (10)	0.0260 (10)	0.0285 (10)	0.0003 (8)	0.0004 (8)	−0.0012 (8)
C3	0.0234 (10)	0.0266 (10)	0.0261 (10)	0.0016 (8)	0.0002 (8)	−0.0027 (8)
C4	0.0333 (12)	0.0328 (11)	0.0234 (10)	−0.0017 (10)	0.0005 (8)	−0.0077 (9)
C5	0.0314 (12)	0.0384 (12)	0.0343 (12)	−0.0035 (10)	0.0044 (9)	−0.0126 (10)
C6	0.0355 (12)	0.0286 (11)	0.0286 (11)	0.0000 (9)	0.0000 (9)	0.0057 (9)
C7	0.0398 (13)	0.0273 (11)	0.0294 (11)	0.0014 (10)	−0.0019 (10)	−0.0004 (9)
C8	0.0317 (12)	0.0322 (12)	0.0296 (11)	0.0003 (10)	−0.0018 (9)	0.0019 (9)
C9	0.0301 (11)	0.0343 (12)	0.0290 (11)	−0.0012 (10)	0.0036 (9)	−0.0052 (9)
N1	0.0282 (9)	0.0283 (9)	0.0234 (9)	−0.0004 (8)	0.0012 (7)	−0.0043 (7)
N2	0.0298 (9)	0.0264 (9)	0.0240 (8)	0.0006 (8)	0.0001 (7)	−0.0002 (7)
O1	0.0579 (11)	0.0434 (10)	0.0286 (8)	0.0069 (8)	0.0023 (7)	0.0058 (7)
O2	0.0662 (12)	0.0343 (9)	0.0397 (9)	0.0182 (9)	0.0040 (8)	0.0020 (8)
O3	0.0539 (11)	0.0401 (10)	0.0368 (9)	0.0166 (8)	0.0043 (8)	−0.0083 (7)
O4	0.0550 (11)	0.0451 (10)	0.0249 (8)	0.0022 (9)	0.0033 (7)	−0.0049 (7)
O5	0.0522 (12)	0.0692 (13)	0.0370 (9)	−0.0241 (10)	0.0166 (8)	−0.0153 (9)
O6	0.0413 (10)	0.0652 (13)	0.0501 (10)	−0.0177 (9)	−0.0020 (8)	−0.0202 (9)
O7	0.0388 (10)	0.0537 (11)	0.0452 (10)	−0.0106 (9)	−0.0016 (8)	0.0126 (9)
O8	0.0497 (11)	0.0645 (12)	0.0296 (9)	−0.0099 (10)	−0.0091 (8)	0.0080 (8)

### Geometric parameters (Å, °)

**Table:**

C1—N1	1.320 (3)	C6—N2	1.467 (3)
C1—N2	1.324 (3)	C6—C7	1.511 (3)
C1—H1	0.9300	C6—H6A	0.9700
C2—C3	1.366 (3)	C6—H6B	0.9700
C2—N2	1.389 (3)	C7—O7	1.199 (3)
C2—C9	1.496 (3)	C7—O8	1.316 (3)
C3—N1	1.385 (3)	C8—O1	1.233 (3)
C3—C8	1.506 (3)	C8—O2	1.263 (3)
C4—N1	1.469 (3)	C9—O4	1.216 (3)
C4—C5	1.511 (3)	C9—O3	1.295 (3)
C4—H4A	0.9700	O2—H3W	1.29 (3)
C4—H4B	0.9700	O3—H3W	1.14 (3)
C5—O6	1.199 (3)	O5—H2W	0.92 (3)
C5—O5	1.315 (3)	O8—H1W	0.93 (4)
			
N1—C1—N2	109.72 (19)	C7—C6—H6B	109.8
N1—C1—H1	125.1	H6A—C6—H6B	108.2
N2—C1—H1	125.1	O7—C7—O8	126.1 (2)
C3—C2—N2	106.45 (17)	O7—C7—C6	123.4 (2)
C3—C2—C9	131.9 (2)	O8—C7—C6	110.53 (19)
N2—C2—C9	121.62 (19)	O1—C8—O2	125.0 (2)
C2—C3—N1	106.91 (18)	O1—C8—C3	118.1 (2)
C2—C3—C8	131.23 (19)	O2—C8—C3	116.90 (19)
N1—C3—C8	121.86 (18)	O4—C9—O3	123.6 (2)
N1—C4—C5	108.45 (17)	O4—C9—C2	120.4 (2)
N1—C4—H4A	110.0	O3—C9—C2	116.01 (19)
C5—C4—H4A	110.0	C1—N1—C3	108.44 (18)
N1—C4—H4B	110.0	C1—N1—C4	122.60 (18)
C5—C4—H4B	110.0	C3—N1—C4	128.64 (17)
H4A—C4—H4B	108.4	C1—N2—C2	108.46 (18)
O6—C5—O5	122.1 (2)	C1—N2—C6	122.56 (19)
O6—C5—C4	122.6 (2)	C2—N2—C6	128.68 (17)
O5—C5—C4	115.28 (19)	C8—O2—H3W	112.5 (14)
N2—C6—C7	109.38 (17)	C9—O3—H3W	112.0 (16)
N2—C6—H6A	109.8	C5—O5—H2W	116 (2)
C7—C6—H6A	109.8	C7—O8—H1W	111 (2)
N2—C6—H6B	109.8		
			
N2—C2—C3—N1	0.3 (2)	N2—C1—N1—C3	1.4 (2)
C9—C2—C3—N1	−177.8 (2)	N2—C1—N1—C4	175.52 (18)
N2—C2—C3—C8	−179.3 (2)	C2—C3—N1—C1	−1.1 (2)
C9—C2—C3—C8	2.5 (4)	C8—C3—N1—C1	178.64 (18)
N1—C4—C5—O6	9.2 (3)	C2—C3—N1—C4	−174.69 (19)
N1—C4—C5—O5	−170.7 (2)	C8—C3—N1—C4	5.0 (3)
N2—C6—C7—O7	−15.1 (3)	C5—C4—N1—C1	−98.9 (2)
N2—C6—C7—O8	166.48 (18)	C5—C4—N1—C3	74.0 (3)
C2—C3—C8—O1	170.6 (2)	N1—C1—N2—C2	−1.2 (2)
N1—C3—C8—O1	−9.0 (3)	N1—C1—N2—C6	−175.48 (18)
C2—C3—C8—O2	−9.5 (3)	C3—C2—N2—C1	0.5 (2)
N1—C3—C8—O2	170.9 (2)	C9—C2—N2—C1	178.88 (18)
C3—C2—C9—O4	−171.7 (2)	C3—C2—N2—C6	174.33 (19)
N2—C2—C9—O4	10.4 (3)	C9—C2—N2—C6	−7.3 (3)
C3—C2—C9—O3	9.6 (3)	C7—C6—N2—C1	90.0 (2)
N2—C2—C9—O3	−168.3 (2)	C7—C6—N2—C2	−83.1 (3)

### Hydrogen-bond geometry (Å, °)

**Table:**

*D*—H···*A*	*D*—H	H···*A*	*D*···*A*	*D*—H···*A*
O3—H3W···O2	1.13 (3)	1.29 (3)	2.426 (3)	177 (3)
C1—H1···O6^i^	0.93	2.47	3.158 (3)	131
C4—H4A···O5^ii^	0.97	2.38	3.311 (3)	160
C4—H4B···O6^i^	0.97	2.37	3.046 (3)	126
C6—H6A···O7^i^	0.97	2.42	3.136 (4)	130
C6—H6B···O8^iii^	0.97	2.44	3.346 (3)	154
O5—H2W···O1^iv^	0.92 (3)	1.67 (3)	2.581 (3)	170 (3)
O8—H1W···O4^v^	0.93 (4)	1.84 (4)	2.710 (3)	155 (3)

Symmetry codes: (i) −*x*+1/2, *y*−1/2, *z*; (ii) *x*−1/2, *y*, −*z*+1/2; (iii) −*x*, −*y*, −*z*+1; (iv) −*x*, *y*−1/2, −*z*+1/2; (v) *x*+1/2, −*y*+1/2, −*z*+1.
